# Effective concentration-based serum pharmacodynamics for antifungal azoles in a murine model of disseminated *Candida albicans* infection

**DOI:** 10.1007/s13318-013-0122-4

**Published:** 2013-03-29

**Authors:** Katsuyuki Maki, Shuji Kaneko

**Affiliations:** 1 Department of Molecular Pharmacology, Graduate School of Pharmaceutical Sciences, Kyoto University, Kyoto, Japan; 2 Present Address: Department of Immunology and Infectious Diseases, Pharmacology Research Laboratories, Astellas Pharma Inc., 21 Miyukigaoka, Tsukuba, Ibaraki 305-8585 Japan

**Keywords:** Candida albicans, Antifungal, Azole, Pharmacodynamics, Serum MIC, Serum antifungal titer

## Abstract

An assessment of the effective in vivo concentrations of antifungal drugs is important in determining their pharmacodynamics, and therefore, their optimal dosage regimen. Here we establish the effective in vivo concentration-based pharmacodynamics of three azole antifungal drugs (fluconazole, itraconazole, and ketoconazole) in a murine model of disseminated *Candida albicans* infection. A key feature of this study was the use of a measure of mycelial (*m*) growth rather than of yeast growth, and pooled mouse sera rather than synthetic media as a growth medium, for determining the minimum inhibitory concentrations (MICs) of azoles for *C. albicans* (denoted serum mMICs). The serum mMIC assay was then used to measure antifungal concentrations and effects as serum antifungal titers in the serum of treated mice. Both serum mMIC and sub-mMIC values reflected the effective in vivo serum concentrations. Supra-mMIC and mMIC effects exhibited equivalent efficacies and were concentration-independent, while the sub-mMIC effect was concentration-dependent. Following administration of the minimum drug dosage that inhibited an increase in mouse kidney fungal burden, the duration periods of these effects were similar for all drugs tested. The average duration of either the mMIC effect including the supra-mMIC effect, the sub-mMIC effect, or the post-antifungal effect (PAFE) were 6.9, 6.5 and 10.6 h, respectively. Our study suggests that the area under the curve for serum drug concentration versus time, between the serum mMIC and the sub-mMIC, and exposure time above the serum sub-mMIC after the mMIC effect, are major pharmacodynamic parameters. These findings have important implications for effective concentration-based pharmacodynamics of fungal infections treated with azoles.

## Introduction

Accurate determination of antifungal pharmacodynamics is important both for successful drug discovery in animal models (Maki et al. 2008) and for improving clinical outcomes with current antifungals by optimizing drug dosage regimens. Previously, non-clinical dose fractionation studies have determined the pharmacodynamic parameters and breakpoints for current antifungals. Defined synthetic media were used in in vitro methods such as the Clinical and Laboratory Standards Institute (CLSI, formerly National Committee for Clinical Laboratory Standards, NCCLS) microdilution reference method (CLSI 2008, NCCLS 2002) and the European Committee on Antimicrobial Susceptibility Testing (EUCAST) agar disk diffusion test (Lass-Flörl et al. 2008) for determining MICs. These dose-based investigations were used to indicate the optimal clinical dose regimen for improving efficacy and for prediction of the clinical outcome against susceptible and resistant pathogens (Lee et al. 2000; Pai et al. 2007; Clancy et al. 2005). Although these MIC data contributed to progress in antifungal therapy, the values do not correspond to the in vivo effective concentrations (Maki et al. 2007, 2008) and accurate pharmacodynamic parameters, such as the time to reach an effective concentration in vivo could not be determined. Furthermore, there are no criteria for determining sub-MIC values using such methods, and the sub-MIC effect cannot be discriminated from the post-antifungal effect (PAFE).

We have previously demonstrated that the use of an ex vivo (mouse serum) assay of mycelial growth (Maki et al. 2006) can be applied to enable direct comparison of the serum concentrations of azoles and the polyene, amphotericin B (Maki et al. 2007) and the in vivo efficacy of echinocandins (Maki et al. 2008) in a mouse model of infection. The use of undiluted serum is important because the bloodstream environment determines most in vivo protein binding of drugs (Hage et al. 2011). Serum may also modulate antifungal activities. For example, in response to serum, there is enhanced expression of the *C. albicans* ergosterol biosynthesis genes, including *ERG11*, which encodes the target protein for azole drugs (Song et al. 2008). The use of mycelial growth as an endpoint for growth inhibition by antifungals is also important because mycelial growth is a key virulence factor of *C. albicans* in the mouse model of disseminated infection, where mycelial formation in kidneys is the major pathology (Saville et al. 2003, 2006). Thus, the above-accumulated evidence indicates that the biological environment during infection with *C.* *albicans* may cause an inconsistent gap between an MIC measured in defined media and the effective in vivo concentration of the antifungal drug.

In this study, we have used an ex vivo (mouse serum) assay of mycelial growth to resolve current issues in antifungal pharmacodynamics, not addressed by the use of synthetic medium-based assays of drug MICs for yeast growth. The effective concentration-based pharmacodynamics of three azole antifungals [which have diverse properties in terms of in vivo elimination half-life, rates of protein binding, and production of active metabolites (Maki et al. 2006, 2007; Saag and Dismukes 1988)] was measured in a mouse candidiasis infection model.

## Materials and methods

### *Candida albicans* strain, culture conditions, and compounds

*Candida albicans* American Type Culture Collection (ATCC) 90028, which is the reference strain recommended in CLSI protocols (NCCLS 2002; CLSI 2008), was used for the in vitro and in vivo assays as described previously (Maki et al. 2006, 2007). The strain was prepared for the assays on Sabouraud dextrose agar containing 2 % (w/v) glucose (Nacalai Tesque, Inc., Kyoto, Japan), 1 % (w/v) polypeptone (Nihon pharmaceutical Co., Ltd., Tokyo, Japan), and 1.5 % (w/v) Bacto-agar (Becton–Dickinson, Sparks, MD, USA) slants at 30 °C. The three azole drugs, fluconazole (Pfizer Japan Inc., Tokyo), itraconazole, and ketoconazole (Janssen Pharmaceutical K. K., Tokyo, Japan), were also prepared as described previously (Maki et al. 2006, 2007). For in vitro assays, itraconazole and ketoconazole were suspended in 10 % dimethyl sulfoxide (Nacalai Tesque, Inc.) as an initial stock. Fluconazole was dissolved in sterile deionized water. For in vivo assays, the drugs were dispersed in 0.5 % methylcellulose (Wako Pure Chemical Industries, Ltd., Osaka, Japan).

### Serum MIC assay for mycelial elongation of *C*. *albicans* (mMIC)

For the in vitro serum susceptibility tests and in vivo efficacy assay, DBA/2 mice (complement component C5-deficient, males, age 6 weeks, Japan SLC, Inc., Shizuoka) were used as described previously (Maki et al. 2007). For the in vitro serum susceptibility assays (Maki et al. 2006, 2007), the 2-[4-(2-Hydroxyethyl)-1-piperazinyl]ethanesulfonic acid (Hepes)-NaOH buffered serum pool (pH 7.4) from normal DBA/2 mice was inoculated with *C. albicans* [1 × 10^4^ colony-forming units (CFU)/ml] and used to dilute each drug and make serial twofold dilutions (100 μl) of the drug in 96 well-microplates. Incubation of 37 °C under 5 % CO_2_ for 14 h in this serum media induces mycelial growth of *C. albicans* ATCC 90028. The endpoint for azole drugs was determined microscopically as the minimum concentration needed for mycelial elongation inhibition (serum mMIC) (Maki et al. 2006, 2007). Serum sub-mMIC was determined as the minimum concentration that ensured an approximate 60 % drug-free mycelial growth. Sub-mMIC was defined as the lower limit of the antifungal activity exhibited by the drug. Microscopy-based assessment of growth was scored using the following scale: −, none or slight germination; ±, blocking of further mycelial elongation (the minimum concentration giving scores of – to ± was defined as the serum mMIC); +, less than or approximately equal to 30 % outgrowth of the drug-free control; ++, less than or approximately equal to 60 % outgrowth of the drug-free control (the minimum concentration giving a score of ++ was defined as the serum sub-mMIC); or +++, growth similar to that of the drug-free control.

### Murine infection model

In vivo antifungal activities of the three azole drugs were determined as described previously (Maki et al. 2007). Briefly, DBA/2 mice (*n* = 10) were intravenously inoculated with *C. albicans* ATCC 90028 (3 × 10^4^ CFU), and each drug was administered orally 1 h later, which was designated as time-point 0. The 90 % effective doses (for survival, ED_90_) of the azole drugs were determined using a Probit analysis for the day on which all the mice in the drug-treated group had died (Maki et al. 2007). The ED_90_ values of the drugs were 0.90 mg/kg for fluconazole, 9.9 mg/kg for itraconazole, and 24.3 mg/kg for ketoconazole. ED_50_ values for the drugs were determined as described previously (fluconazole: 0.52 mg/kg, itraconazole: 4.8 mg/kg, ketoconazole: 17.0 mg/kg) (Maki et al. 2007). For the microbiological assays, the fungal burden in the kidneys of the infected mice was determined as described previously (Maki et al. 2007). A pair of kidneys were homogenized in 0.9 % saline using a Polytron^®^ homogenizer (Kinematica AG, Littau, Switzerland). The fungal burden of *C. albicans* from the homogenate was determined on Sabouraud dextrose agar plates as CFU/organ.

### Determination of drug serum concentration

Serum drug concentration was determined as the serum antifungal titer by means of a limiting dilution assay, which was carried out under the same conditions used for the serum mMIC assay (Maki et al. 2006, 2007). Serum samples from the *C. albicans*-infected and drug-treated DBA/2 mice were buffered with Hepes–NaOH (pH 7.4) and inoculated directly with *C. albicans* (1 × 10^4^ CFU/ml) as the initial dosage. Serial twofold dilutions were made using pooled drug-free DBA/2 mouse serum that was buffered with Hepes–NaOH (pH 7.4) and inoculated with *C. albicans* (1 × 10^4^ CFU/ml) as a diluent, and dispensed into a microplate (100 μl aliquots). After incubation for 14 h at 37 °C under 5 % CO_2_, the serum antifungal titer of the serum samples was defined as the reciprocal of the highest dilution of serum that achieved the level of growth inhibition that corresponded to the endpoint criteria of the serum mMIC assay. Thus, an antifungal titer of 1 corresponded to the serum mMIC.

In serum samples from each mouse, when the inhibitory activity was comparable to the sub-mMIC, the antifungal activity was defined as the detection limit of the antifungal titer. When the antifungal titer of the initial serum was less than 1, the sub-inhibitory activities were plotted according to the scoring criteria described above: 0.5 for +; 0.25 for ++ (sub-mMIC); and 0.125 for +++. Itraconazole is converted into an active metabolite in mice, therefore, serum antifungal titer includes the activity of the metabolite (Maki et al. 2006).

### Pharmacokinetic analysis

Serum concentrations of the drugs were determined using the serum antifungal titers (Maki et al. 2006, 2007). Pharmacokinetic parameters were calculated according to the one-compartment model.

$$ C = C^{ 0} \cdot {\text{exp(}} - K \cdot t ) $$

where *C* is the serum concentration at time *t*. *K* is the elimination rate constant. *C* ^0^ is the extrapolated serum concentration at time 0. The time to maintain serum mMIC and sub-mMIC effects was also determined from the above equation for antifungal titer (Table 1). The AUC determined in this study was also calculated by the trapezoidal rule using antifungal titers-time curve and cut out antifungal titer-time area below serum sub-mMIC. The AUC is illustrated as the area, which has antifungal activity against mycelial elongation as defined in this study.

**Table 1:** Microbiological pharmacodynamic parameters of azole drugs administered at ED_90_ doses to *C. albicans* ATCC 90028-infected mice

	Maximum titer^a^	mMIC effect duration (h)^b^	Sub-mMIC effect duration (h)^c^	PAFE duration (h)^d^	Total AUC_0–24 h_ (serum antifungal titer h)^e^	AUC_0–24 h_ below mMIC (serum antifungal titer h)^f^
Fluconazole	2.0 (1)^g^	6.0 (25.0)^h^	8.0	10	9.1 (1)^g^	7.5 (1)^g^
Itraconazole^i^	2.0 (1.0)	9.0 (37.5)	4.5	10.5	10.4 (1.1)	8.1 (1.1)
Ketoconazole	26.9 (13.5)	5.7 (23.8)	7.1	11.2	44.5 (4.9)	5.6 (0.75)
Average duration time (h) [proportion of 24 h (%)]^j^	ND	6.9 (28.8)^j^	6.5 (27.2)	10.6 (44.0)	ND	ND

Pharmacodynamic parameters of the ED_90_ doses were calculated using the serum antifungal titer as the serum concentration. *ND* not determined. Parenthesis shows average proportion of the effects during 24 h (%)

^a^Titer maximum (serum antifungal titer, or ×serum mMIC), i.e., the peak serum antifungal titer after a single administration of the drug

^b^Includes supra-mMIC effect, the time during which the serum antifungal titer is at or above a value of 1 (serum mMIC) after a single administration of the drug

^c^Occurs after the mMIC effect, the time for which the serum antifungal titer was between a value of 1 and its detection limit (serum sub-mMIC)

^d^Up to 24 h after administration, the time after the serum antifungal titer fell below its detection limit (serum sub-mMIC)

^e^The AUC_0–24 h_ at or above serum sub-mMIC from the serum antifungal titer-time curve, AUC_0–24 h_ when drug has antifungal effects (serum mMIC or sub-mMIC effects)

^f^The AUC_0–24 h_ between the serum mMIC and the sub-mMIC for the serum antifungal titer-time curve

^g^Ratio of the value for fluconazole

^h^Proportion of 24 h for mMIC effect of the drugs (%)

^i^Serum antifungal titer of itraconazole includes activity of an active metabolite (Maki et al. 2006)

^j^Average duration time during 24 h for each effect of the three drugs

### Statistical analysis

The correlation between serum antifungal titer and fungal burden in kidneys was examined using the two-phase decay formula in Prism 5 for Windows (GraphPad Software Inc. San Diego, CA, USA). The in vivo anti-kidney fungal burden data were compared using the Mann–Whitney *U* test. A *P* value of <0.05 was considered statistically significant.

## Results

### Activities of azole antifungals against *C. albicans*

For in vitro antifungal activities of fluconazole, itraconazole, and ketoconazole against *C. albicans* ATCC 90028, the serum mMIC and sub-mMIC were determined using pooled mouse sera and inhibition of *C. albicans* mycelia elongation. Serum mMIC values and sub-mMIC values of the drugs were as follows: fluconazole, 0.5 and 0.13 μg/ml; itraconazole, 1 and 0.25 μg/ml; and ketoconazole, 0.5 and 0.13 μg/ml. These mMIC and sub-mMIC values were included as the effective concentrations in the in vivo pharmacodynamic investigation for our study (Maki et al. 2007).

### Relationship between antifungal effects and pharmacodynamic parameters

**Fig. 1:**
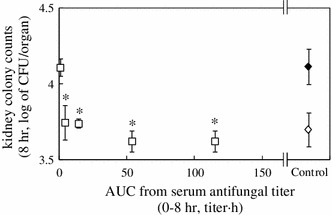
Relationship between AUC_0–8 h_ and the inhibitory effects of fluconazole on kidney fungal burden. The drug was administered orally to DBA/2 mice 1 h after fungal inoculation. The dosages of drugs were ×1/3 ED_50_, ×1 ED_50_, ×3 ED_50_ ×10 ED_50_, and ×30 ED_50_. AUC up to 8 h of the dosages and anti-kidney fungal burden of *C. albican*s at 8 h were determined. AUC_0–8 h_ was determined only from active concentrations of the drug at concentrations at or above serum sub-mMIC. □, ED_50_-based doses of fluconazole at 8 h; ◇, vehicle-treated control at time 0 (5.0 × 10^3^ CFU/organ); ◆, vehicle-treated control at 8 h. Each *point* represents the data from four mice and the values shown are the mean ± standard deviation. Differences between fugal burdens of each doses and at 0 time were analyzed statistically. The *asterisks* indicate *P* values of >0.05

Figure 1 illustrates the relationship between the antifungal activity of fluconazole and AUC above sub-mMIC for 8 h (AUC_0–8 h_) after drug administration in the *C. albicans* infection mouse model. That is, the AUC_0–8 h_ indicated the AUC of antifungal activity at or above the serum sub-effective concentration. Antifungal activity is determined as anti-kidney fungal burden activity. At the higher AUC_0–8 h_ of the curve (Fig. 1), the effect reaches a maximum and is independent of AUC_0–8 h_. As shown in Fig. 1, *C. albicans* cells are recovered even at the highest dose (×30 ED_50_ 8 h), at values equivalent to the 0 time-point CFU value. This reflects the fact that azole drugs are fungistatic, and therefore, the inoculum appears to survive in the presence of the drug. Furthermore, at AUC values of 50 or above, the kidney colony counts did not decrease, indicating that antifungal activity is at a steady state.

### Determination of the minimum dosage of an antifungal agent that inhibits an increase in kidney fungal burden after 24 h

**Fig. 2:**
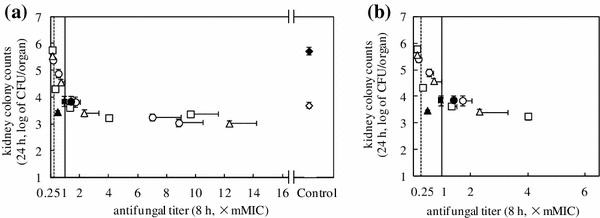
Relationship between serum antifungal titers and the inhibitory effects of fluconazole, itraconazole, and ketoconazole on kidney fungal burden. Azole doses were administered orally to DBA/2 mice 1 h (time 0 in the figure) after fungal inoculation. **a** The relationship between serum antifungal titer after 8 h and the inhibitory effect of the drug against the kidney fungal burden after 24 h was determined. ◇, vehicle-treated control at time 0 (5.0 × 10^3^ CFU/organ); ◆, vehicle-treated control after 24 h (5.4 × 10^5^ CFU/organ); □■, fluconazole; ○●, itraconazole; △▲, ketoconazole. The *closed symbols* (■, ●, ▲) represent plots of the drugs administered at the ED_90_ doses. The *vertical solid line* denotes an antifungal titer of 1 (the serum mMIC). The *dotted vertical line* denotes the detection limit of the antifungal titer (the serum sub-mMIC). Serum antifungal titer of itraconazole includes the activity of an active metabolite (Maki et al. 2006). Co-efficiency of the combined plots of the three azoles: *r* ^*2*^ = 0.88. Each plot includes data from four mice and the values shown are the mean ± standard deviation. Plots other than the ED_90_ plots are adopted from a previous report (Maki et al. 2007). **b** An expanded view showing data obtained at antifungal titers between 0.25 and 6

On the assumption that antifungal drugs are administered once daily, the minimum dosages that inhibited an increase in kidney fungal burden at 24 h in the *C. albicans* mouse infection model were determined from the serum antifungal titer-kidney fungal burden curve (Fig. 2). A clear correlation between in vivo efficacy and serum antifungal titer was observed for the three azole drugs, as reported previously (Maki et al. 2007) using a two-phase exponential decay analysis (*r* ^2^ = 0.93). In this study, we found that the antifungal activities for the ED_90_ dosages of the azoles were in the proximity of the inflection point of the biphasic curve (Fig. 2). The ED_90_ dosages were defined as the minimum dosages needed to inhibit an increase in the kidney fungal burden, 24 h after drug administration.

### Determination of ED_90_ dosage pharmacodynamics

**Fig. 3:**
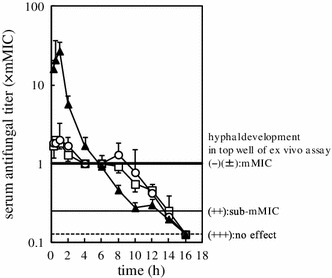
Serum antifungal titers in serum samples from mice administered ED_90_ doses of fluconazole, itraconazole, and ketoconazole. ED_90_ doses of azoles were administered orally to DBA/2 mice 1 h (time 0 in the figure) after fungal inoculation, and the antifungal titers were determined every 2 h thereafter. The *bold line* denotes an antifungal titer of 1 (the serum mMIC). The *solid line* denotes the detection limit of the serum antifungal titer (the serum sub-mMIC). Serum antifungal titer of itraconazole includes the activity of an active metabolite (Maki et al. 2006). □, fluconazole; ○, itraconazole; ▲, ketoconazole; each *point* represents the data from four mice and the values shown are the mean ± standard deviation

To investigate the antifungal effects in terms of the fungal burden in the kidneys of infected mice over the 24 h period after the administration of the antifungal drugs, ED_90_ dosages were administered to *C. albicans*-infected mice and serum antifungal titers were determined (Fig. 3). The serum antifungal titers from drug-administered mice reflected the impact of the drug on fungal counts in the kidneys (Maki et al. 2007). Thus, it was possible to determine the antifungal effects of the drugs against the kidney fungal burdens using the serum antifungal titer method. The peak serum antifungal titers (maximum titer) at the ED_90_ dosages differed by as much as 13.5-fold (Fig. 3; Table 1). The time for the supra-mMIC and serum mMIC effects ranged from 5.7 to 9 h (Table 1). The duration of serum sub-mMIC effects, which corresponded to the antifungal activity between serum mMIC and the sub-mMIC, was sustained for up to 12.8–14 h (Fig. 3; Table 1). PAFEs of the drugs were detected from those times to 24-h post-administration. Thus, the time in which drug concentrations are on or above serum sub-mMIC was considered to be an important pharmacodynamic parameter for each of the azole drugs studied.

Exposure to higher concentrations than the serum mMIC did not further contribute to the drug having a stronger inhibitory effect (Fig. 2); therefore, serum antifungal activities at concentrations above the mMIC should not be considered. Although the values for the total area under the serum drug concentration versus time curve (AUC_0–24 h_) for the serum antifungal titers of the three drugs displayed as much as 4.9-fold differences, the AUC_0–24 h_ at or below the mMIC were less variant among the azoles (Table 1). The important pharmacodynamic parameters for the azole ED_90_ dosages were the AUC_0–24 h_ between the serum mMIC and the sub-mMIC, as well as the exposure time above the serum sub-mMIC after the mMIC effect (but not the supra-mMIC effect).

## Discussion

Antifungal pharmacodynamics can determine the relationship between drug exposure and outcome (Andes 2003; Theuretzbacher et al. 2006), with drug exposure being influenced by drug concentration and exposure time. A previous in vivo time kill assay for azoles and amphotericin B (Maki et al. 2007) showed that the effective in vivo concentration was a determinant of antifungal effects. Exposure time, which includes the time from inhibition of the intracellular target molecule to expression of fungal growth inhibition, maintained these antifungal effects, yet it was not a determinant of pharmacodynamic parameters. Thus, the main pharmacodynamics of these drugs was focused on the relationships between concentration and effect. The serum mMIC and sub-mMIC values reflected the effective and sub-effective in vivo serum concentrations, respectively, with respect to the inhibitory activity of the azole drugs against the fungal burden in the kidneys in the disseminated infection model (Maki et al. 2007). Using serum mMIC and sub-mMIC, the in vivo serum supra-mMIC, mMIC, and sub-mMIC effects, and the PAFE were successfully defined. The data showed that the serum mMIC was equivalent to the saturation concentration for in vivo efficacy. Serum supra-mMIC and mMIC effects were concentration-independent, whereas the sub-mMIC effect was concentration-dependent (Maki et al. 2007). Thus, the tested drugs probably express multiple effects associated with the rapid change in drug concentration after administration in vivo.

In this study, effective concentration-based serum pharmacodynamics was investigated from two points of view: improvement of efficacy (as a general concept) and identification of the key features for daily minimum dosage of ED_90_ (for investigating in vivo antifungal mechanisms). As to pharmacodynamics for improvement of efficacy, if a drug reaches the maximum plateau phase within a clinically achievable level, the time in which drug concentrations coincide or exceed the saturation concentration (serum mMIC) should be taken as the main pharmacodynamic parameter regardless of its mode of action and structure. This study (Table 1; Figs. 2 and 3), although showing that itraconazole which is converted into an active metabolite in mouse (Maki et al. 2006), gave the longest mMIC effect duration (Table 1), suggests that any of three azole drugs, fluconazole, itraconazole, and ketoconazole showed saturation phase (the main supra-mMIC and mMIC effects) after administration of higher doses than ED_90_. However, this is contrary to previously published results, which showed that the pharmacodynamic parameters were assigned as a single property with each drug (Andes 2003). The occurrence of a maximum effect of three drugs (Fig. 2) reflects the fact that azole drugs did not show an increase in antifungal effects at drug concentrations above the serum mMIC in the infection model (Maki et al. 2007). This saturation phase of AUC is important for effective concentration-based pharmacodynamics in serum.

Our study successfully identified multiple novel pharmacodynamic features of the azole drugs when the minimum dosages of azoles were administered. Changes in the kinetics of antifungal effects for our study were detected based on serum antifungal titers, which estimated the multiple inhibitory effects of the drug against fungal infection in the kidney (Maki et al. 2007). Antifungal titer assays indicated that the sub-mMIC effects, which were evident after the main supra-mMIC and serum mMIC effects, finished 12.8–14 h after drug administration. The supra-mMIC effect was comparable to the serum mMIC effect, and was involved in the serum mMIC effect. We therefore suggest that the exposure time above the serum sub-mMIC following the mMIC effect, and the AUC_0–24 h_ between the serum mMIC and the sub-mMIC are important pharmacodynamic parameters for azole ED_90_ dosage.

Taking the AUC and the maximum concentration-independent effects of azoles into consideration (Fig. 1) (Andes 2003; Klepser et al. 1997; Maki et al. 2007; Theuretzbacher et al. 2006), the time above serum mMIC should be given priority as the main pharmacodynamic parameter. As an example, the magnitude of time above serum mMIC for fluconazole in this study was 25.0 % (the percentage of time per 24 h that the concentration exceeded the serum mMIC, Table 1). Recent non-clinical dose fractionation studies suggested that the main in vivo pharmacodynamic parameter for azole drugs was AUC (Andes and van Ogtrop 1999; Andes 2003; Andes et al. 2003a, b, Andes et al. 2004; Louie et al. 1998; Theuretzbacher et al. 2006). However, AUC is a concentration-associated factor and does not support the finding (Andes 2003; Klepser et al. 1997; Maki et al. 2007; Theuretzbacher et al. 2006) that the maximum antifungal effect of azoles is concentration independent.

The average duration of the serum mMIC effect was only one-third (28.8 %) of the 24 h time-course used to assess inhibition of fungal infection in the kidney (Table 1). The component effects (sub-mMIC effect and PAFE) sustained the serum mMIC effect during the remaining two-thirds of the 24 h period. Unexpectedly, the average PAFE (44.0 %, Table 1) maintained a longer action than both the mMIC and sub-mMIC effects in this infection model (Table 1). Previous reports of the in vitro PAFE for azole drugs have shown divergent results, with presence or absence of PAFE reported because of variations in assay methodology (Ernst et al. 2000; García et al. 1999; Minguez et al. 1994; Zhanel et al. 2001). Although previous in vivo investigations using MICs determined in defined synthetic media reported the PAFE of antifungals as criteria of the MIC but not sub-MIC, only the sub-MIC and PAFE were considered together in these studies (Andes and van Ogtrop 1999; Andes 2003). Our study has established, for the first time, that the component effects influence in vivo efficacy, as well as the main supra-mMIC and serum mMIC effects in this particular infection model.

We have established here the effective concentration-based serum pharmacodynamics for single dosing of three azole drugs. Future studies would include investigating the effects of drugs using repeated dosing in a clinically relevant infection model. Determination of the in vivo effective concentrations, using serum-based assays, would be critical for not only clinical efficacy, but also for reducing the risk of adverse effects and to improve the success rate in drug discovery.

## References

[CR1] Andes D (2003). In vivo pharmacodynamics of antifungal drugs in treatment of candidiasis. Antimicrob Agents Chemother.

[CR2] Andes D, van Ogtrop M (1999). Characterization and quantitation of the pharmacodynamics of fluconazole in a neutropenic murine disseminated candidiasis infection model. Antimicrob Agents Chemother.

[CR3] Andes D, Marchillo K, Stamstad T, Conklin R (2003). In vivo pharmacokinetics and pharmacodynamics of a new triazole, voriconazole, in a murine candidiasis model. Antimicrob Agents Chemother.

[CR4] Andes D, Marchillo K, Stamstad T, Conklin R (2003). In vivo pharmacodynamics of a new triazole, ravuconazole, in a murine candidiasis model. Antimicrob Agents Chemother.

[CR5] Andes D, Marchillo K, Conklin R, Krishna G, Ezzet F, Cacciapuoti A, Loebenberg D (2004). Pharmacodynamics of a new triazole, posaconazole, in a murine model of disseminated candidiasis. Antimicrob Agents Chemother.

[CR6] Clancy CJ, Yu VL, Morris AJ, Snydman DR, Nguyen MH (2005). Fluconazole MIC and the fluconazole dose/MIC ratio correlate with therapeutic response among patients with candidemia. Antimicrob Agents Chemother.

[CR7] Clinical and Laboratory Standards Institute, Wayne, PA (2008) Reference method for broth dilution antifungal susceptibility testing of yeasts: Approved standard, 3rd edn, M27–A3

[CR8] Ernst EJ, Klepser M, Pfaller MA (2000). Postantifungal effects of echinocandin, azole, and polyene antifungal agents against *Candida albicans* and *Cryptococcus neoformans*. Antimicrob Agents Chemother.

[CR9] García MT, Llorente MT, Lima JE, Mínguez F, Del Moral F, Prieto J (1999). Activity of voriconazole: post-antifungal effect, effects of low concentrations and of pretreatment on the susceptibility of *Candida**albicans* to leucocytes. Scand J Infect Dis.

[CR10] Hage DS, Anguizola J, Barnaby O, Jackson A, Yoo MJ, Papastavros E, Pfaunmiller E, Sobansky M, Tong Z (2011). Characterization of drug interactions with serum proteins by using high-performance affinity chromatography. Curr Drug Metab.

[CR11] Klepser ME, Wolfe EJ, Jones RN, Nightingale CH, Pfaller MA (1997). Antifungal pharmacodynamic characteristics of fluconazole and amphotericin B tested against *Candida albicans*. Antimicrob Agents Chemother.

[CR12] Lass-Flörl C, Mayr A, Perkhofer S, Hinterberger G, Hausdorfer J, Speth C, Fille M (2008). Activities of antifungal agents against yeasts and filamentous fungi: assessment according to the methodology of the European Committee on antimicrobial susceptibility testing. Antimicrob Agents Chemother.

[CR13] Lee SC, Fung CP, Huang JS, Tsai CJ, Chen KS, Chen HY, Lee N, See LC, Shieh WB (2000). Clinical correlates of antifungal macrodilution susceptibility test results for non-AIDS patients with severe *Candida* infections treated with fluconazole. Antimicrob Agents Chemother.

[CR14] Louie A, Drusano GL, Banerjee P, Liu QF, Liu W, Kaw P, Shayegani M, Taber H, Miller MH (1998). Pharmacodynamics of fluconazole in a murine model of systemic candidiasis. Antimicrob Agents Chemother.

[CR15] Maki K, Watabe E, Iguchi Y, Nakamura H, Tomishima M, Ohki H, Yamada A, Matsumoto S, Ikeda F, Tawara S, Mutoh S (2006). Determination of antifungal activities in serum samples from mice treated with different antifungal drugs allows detection of an active metabolite of itraconazole. Microbiol Immunol.

[CR16] Maki K, Holmes AR, Watabe E, Iguchi Y, Matsumoto S, Ikeda F, Tawara S, Mutoh S (2007). Direct comparison of the pharmacodynamics of four antifungal drugs in a mouse model of disseminated candidiasis using microbiological assays of serum drug concentrations. Microbiol Immunol.

[CR17] Maki K, Matsumoto S, Watabe E, Iguchi Y, Tomishima M, Ohki H, Yamada A, Ikeda F, Tawara S, Mutoh S (2008). Use of a serum-based antifungal susceptibility assay to predict the in vivo efficacy of novel echinocandin compounds. Microbiol Immunol.

[CR18] Minguez F, Chiu ML, Lima JE, Ñique R, Prieto J (1994). Activity of fluconazole: postantifungal effect, effects of low concentrations and of pretreatment on the susceptibility of *Candida albicans* to leucocytes. J Antimicrob Chemother.

[CR19] National Committee for Clinical Laboratory Standards, Wayne, PA (2002) Reference method for broth dilution antifungal susceptibility testing of yeasts: Approved standard. NCCLS document M27–A2

[CR20] Pai MP, Turpin RS, Garey KW (2007). Association of fluconazole area under the concentration-time curve/MIC and dose/MIC ratios with mortality in nonneutropenic patients with candidemia. Antimicrob Agents Chemother.

[CR21] Saag MS, Dismukes WE (1988). Azole antifungal agents: emphasis on new triazoles. Antimicrob Agents Chemother.

[CR22] Saville SP, Lazzell AL, Monteagudo C, Lopez-Ribot JL (2003). Engineered control of cell morphology in vivo reveals distinct roles for yeast and filamentous forms of *Candida albicans* during infection. Eukaryot Cell.

[CR23] Saville SP, Lazzell AL, Bryant AP, Fretzen A, Monreal A, Solberg EO, Monteagudo C, Lopez-Ribot JL, Milne GT (2006). Inhibition of filamentation can be used to treat disseminated candidiasis. Antimicrob Agents Chemother.

[CR24] Song Y, Cheon SA, Lee KE, Lee SY, Lee BK, Oh DB, Kang HA, Kim JY (2008). Role of the RAM network in cell polarity and hyphal morphogenesis in *Candida albicans*. Mol Biol Cell.

[CR25] Theuretzbacher U, Ihle F, Derendorf H (2006). Pharmacokinetic/pharmacodynamic profile of voriconazole. Clin Pharmacokinet.

[CR26] Zhanel GG, Saunders DG, Hoban DJ, Karlowsky JA (2001). Influence of human serum on antifungal pharmacodynamics with *Candida albicans*. Antimicrob Agents Chemother.

